# Influence of Organic Salts on Molecular Interactions, Film Performance, and Antimicrobial Activity of TPS/PBAT Blown Films

**DOI:** 10.3390/foods15071148

**Published:** 2026-03-27

**Authors:** Vannet Roschhuk, Phanwipa Wongphan, Yeyen Laorenza, Phatthranit Klinmalai, Nathdanai Harnkarnsujarit

**Affiliations:** 1 Department of Packaging and Materials Technology, Faculty of Agro-Industry, Kasetsart University, 50 Ngam Wong Wan Rd., Latyao, Chatuchak, Bangkok 10900, Thailand; roschhukvannetroschhuk.v@ku.th (V.R.); phanwipa.w@ku.th (P.W.); yeyen.la@ku.th (Y.L.); 2 Faculty of Agro-Industry, Chiang Mai University, Samut Sakhon 74000, Thailand; 3 Center for Advanced Studies for Agriculture and Food (CASAF), Kasetsart University Institute for Advanced Studies (KUIAS), Kasetsart University, 50 Ngam Wong Wan Rd., Latyao, Chatuchak, Bangkok 10900, Thailand

**Keywords:** thermoplastic starch, organic salts, sodium citrate, calcium citrate, calcium lactate

## Abstract

This study investigates the effects of organic salts, including sodium citrate (SC), calcium citrate (CC), and calcium lactate (CL), on the structure–property–function relationships of thermoplastic starch/poly(butylene adipate-co-terephthalate) (TPS/PBAT) films for active packaging applications. TPS incorporated with organic salts was prepared via twin-screw extrusion, blended with PBAT, and further processed into blown films. The films were systematically characterized using ^1^H NMR, FTIR, and SEM, together with optical, mechanical, water vapor permeability, and antimicrobial evaluations against *Staphylococcus aureus*. The results revealed that SC primarily modulated hydrogen-bonding interactions within the starch matrix, resulting in improved structural homogeneity, balanced mechanical properties, and the highest antimicrobial activity among all formulations. In contrast, CL and CC promoted ionic crosslinking through Ca^2+^–starch interactions, leading to increased stiffness and Young’s modulus but reduced polymer chain mobility and limited release of active species, particularly in CC-containing systems. These differences in molecular interactions were consistent with variations in film microstructure, where SC-containing films exhibited more uniform morphologies, while calcium-based systems showed denser but less permeable structures. Furthermore, films containing SC and CL at appropriate concentrations achieved a favorable balance between transparency, water vapor barrier properties, and antimicrobial performance. Overall, this study provides new mechanistic insights into how monovalent and divalent organic salts govern intermolecular interactions, microstructure, and functional performance in TPS/PBAT systems. The findings highlight the critical role of additive type and concentration in designing biodegradable active packaging materials with tunable mechanical, barrier, and antimicrobial properties.

## 1. Introduction

The packaging industry is currently facing major environmental challenges associated with the extensive use of petroleum-based plastics, which generate persistent waste and pose long-term risks to ecosystems. As a result, the development of packaging materials has increasingly focused on biodegradable alternatives derived from renewable resources, while maintaining the functional properties required for practical applications, such as mechanical strength and barrier performance against moisture and gases [1,2,3]. In parallel, packaging concepts have evolved from passive barrier systems toward active packaging, in which packaging materials interact with their surrounding environment through mechanisms such as moisture regulation, radiation shielding, microbial inhibition, or surface modification [4,5,6]. These functionalities are intended to enhance product stability and reduce quality deterioration during storage. Consequently, there is growing interest in designing biodegradable packaging materials that integrate both structural integrity and functional performance to address sustainability and food preservation demands [5,7,8,9,10].

Thermoplastic starch (TPS) is one of the most extensively studied biodegradable materials due to its renewable origin, low cost, biodegradability, and compatibility with food systems. However, TPS suffers from inherent limitations, including poor mechanical strength and weak barrier properties, particularly its high sensitivity to moisture and environmental conditions, which restrict its direct use in commercial food packaging [11,12]. To overcome these limitations, TPS is often blended with other biodegradable polymers that provide improved flexibility and moisture resistance [3,13,14]. Poly(butylene adipate-co-terephthalate) (PBAT) is a biodegradable polyester with excellent flexibility, good mechanical properties, and high processability using conventional industrial techniques such as extrusion and blown-film processing. Blending TPS with PBAT can improve film flexibility and mechanical performance while reducing material costs and increasing the biobased content. Nevertheless, the strong polarity difference between TPS, which contains abundant hydroxyl groups, and the relatively hydrophobic PBAT matrix often leads to phase incompatibility and non-uniform dispersion of TPS, adversely affecting the mechanical and barrier properties of the resulting films. A common strategy to improve the structure and performance of TPS/PBAT systems is the incorporation of additives capable of forming molecular-level interactions with starch and/or PBAT [6,8,11,13,14,15]. Among these additives, organic salts are of particular interest because their carboxylate groups can participate in hydrogen bonding or ionic interactions with polymer functional groups [16,17,18]. In addition, certain organic salts, such as citrate and lactate salts, are widely accepted as food-grade compounds and have been reported to exhibit antimicrobial activity through mechanisms involving local pH reduction or disruption of ionic balance within microbial cells.

Among food-grade organic salts, sodium citrate (SC) and calcium citrate (CC) are commonly used as acidity regulators and chelating agents, while calcium lactate (CL) is widely applied as a soluble calcium source with sustained ion release capability. Differences in metal cations (Na^+^ versus Ca^2+^) and anion structures (citrate versus lactate) strongly influence their interaction mechanisms with starch [16,17,19]. Sodium ions generally promote hydrogen-bonding interactions and improved dispersion within polymer matrices, whereas divalent calcium ions can induce ionic crosslinking between polymer chains, leading to increased structural density but restricted chain mobility [9,19].

Previous studies have extensively explored extrusion-based processing of TPS and TPS/PBAT films, demonstrating their effectiveness in producing biodegradable films with improved processability and mechanical performance [20,21,22,23,24]. However, most of these studies have primarily focused on plasticizers, compatibilizers, or nanofillers, while comparatively limited attention has been given to the role of low-molecular-weight functional additives, such as food-grade organic salts, in modifying intermolecular interactions and functional properties of TPS-based systems. In addition, although certain organic salts, including citrate and lactate compounds, have been individually investigated in polymer systems, systematic comparisons of different salt types, particularly with respect to monovalent (Na^+^) and divalent (Ca^2+^) cations and their structure–property–function relationships in TPS/PBAT films, remain scarce. The combined effects of salt chemistry, ionic interactions, and release behavior on both material performance and antimicrobial functionality have not been fully elucidated.

Therefore, the objective of this study was to systematically investigate the effects of different organic salts, namely sodium citrate (SC), calcium citrate (CC), and calcium lactate (CL), at concentrations ranging from 0 to 7 wt%, on the structural characteristics, mechanical properties, optical behavior, water vapor barrier performance, and antimicrobial activity against Staphylococcus aureus of TPS/PBAT films. Particular emphasis was placed on elucidating the structure–property–function relationships governed by salt type (monovalent versus divalent cations) and interaction mechanisms within the polymer matrix. This study provides new mechanistic insights into how organic salt chemistry can be used to tailor intermolecular interactions, microstructure, and functional performance of biodegradable active packaging films.

## 2. Materials and Methods

### 2.1. Preparation of PBAT/TPS Films Incorporating Organic Salts

Native cassava starch (Siam Modified Starch Co., Ltd., Pathumthani, Thailand) was dried in a hot-air oven at 50 ± 2 °C overnight. The dried starch was mixed with glycerol (Patum Vegetable Oil Co., Ltd., Pathumthani, Thailand) at a starch-to-glycerol ratio of 100:35 (*w*/*w*) using a dough mixer (SC-236A, Stelang, Junan, China) for 10 min. Organic salts, including sodium citrate (Na_3_C_6_H_5_O_7_), calcium citrate (Ca_3_(C_6_H_5_O_7_)_2_), and calcium lactate (C_6_H_10_CaO_6_), were purchased from Chanjao Longevity Co., Ltd., Bangkok, Thailand, and incorporated at concentrations of 0, 1, 2, 3, 5, and 7% (*w*/*w*, based on the total film formulation).

Each organic salt was premixed with glycerol and heated on a hot-plate stirrer at 90 °C until completely dissolved to obtain a homogeneous plasticizer–salt solution. The solution was subsequently blended with the starch for an additional 10 min using the dough mixer. The mixtures were processed into thermoplastic starch (TPS) and TPS containing organic salts using a co-rotating twin-screw extruder (L/D 40; screw diameter 20 mm; Labtech Engineering, Samut Prakan, Thailand). The barrel temperature profile ranged from 110 to 150 °C, and the screw speed was maintained at 180 rpm. The extrudate was cooled in ambient air and pelletized into 2.5 mm granules.

TPS or salt-incorporated TPS pellets were compounded with poly(butylene adipate-co-terephthalate) (PBAT; Ecoflex^®^ F Blend C1200, BASF, Ludwigshafen, Germany) at a PBAT/TPS weight ratio of 60/40 using a twin-screw extruder operated at 100–150 °C and 180 rpm. The resulting PBAT/TPS blend was pelletized to 2.5 mm granules and stored in airtight polyethylene bags.

**Figure 1:**
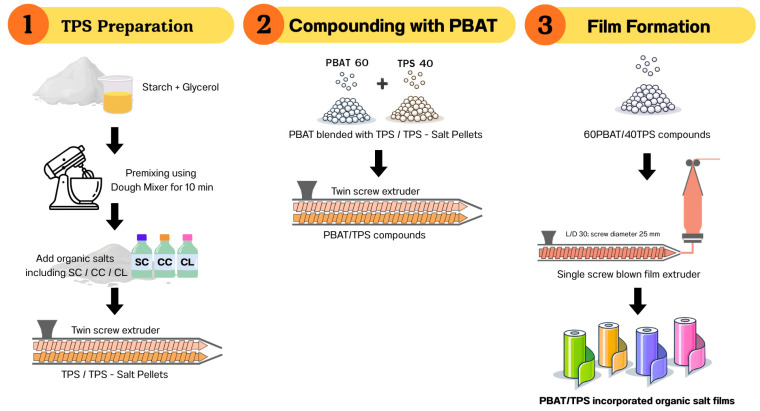
Schematic flowchart for the preparation of TPS/PBAT films incorporating organic salts.

Prior to film formation, the PBAT/TPS pellets were dried overnight at 50 °C in a hot-air oven. Films were produced using a single-screw blown-film extruder (L/D 30; screw diameter 25 mm; Labtech Engineering, Samut Prakan, Thailand) with a temperature gradient of 145–155 °C from the hopper to the die. The screw speed was adjusted to 30–40 rpm, and the nip-roll speed to 2.6–3.0 rpm. The resulting films were stored in Ziploc bags at room temperature until further analysis. The schematic flowchart is shown in Figure 1.

### 2.2. Proton Nuclear Magnetic Resonance (^1^H-NMR) Analysis

^1^H NMR spectra were recorded using an Ascend^TM^ 600/Avance III HD spectrometer (Bruker, Zurich, Switzerland). Selected salt-incorporated TPS pellets containing 2, 5, and 7 wt% SL were dissolved in deuterated dimethyl sulfoxide (DMSO-d_6_) prior to analysis. Chemical shifts were reported in parts per million (ppm) relative to tetramethylsilane (TMS) as the internal reference.

### 2.3. Fourier-Transform Infrared Spectroscopy (FTIR)

The chemical structure of the film samples was analyzed using Fourier-transform infrared (FTIR) spectroscopy. Measurements were performed on a Bruker Tensor 27 FT-IR spectrometer (Bruker Optik GmbH, Leipzig, Germany) operated with spectrum OPUS 6.5 software.. Spectra were collected in Attenuated Total Reflectance (ATR) mode at a resolution of 4 cm^−1^ with 64 accumulated scans. Each spectrum was recorded over the wavenumber range of 4000–500 cm^−1^ using air as the background. Triplicate spectra were obtained and averaged for analysis.

### 2.4. Scanning Electron Microscopy (SEM)

The surface and cross-sectional morphologies of the film samples were examined using a scanning electron microscope (JEOL JSM-IT300, JEOL Ltd., Tokyo, Japan). Cross-sectional specimens were prepared by cryogenic fracturing in liquid nitrogen, followed by desiccation over silica gel to remove residual moisture. The fractured samples were mounted on aluminum stubs and sputter-coated with a thin layer of gold using a Quorum Polaron SC7620 coater (Quorum Technologies, Lewes, UK) to enhance surface conductivity. Morphological observations were conducted at an accelerating voltage of 2.0 kV and a magnification of 1000×.

### 2.5. Light Transmission

Light transmission of the film samples was measured using a UV–Vis spectrophotometer (Evolution 300, Thermo Scientific, Waltham, MA, USA). Spectra were recorded over a wavelength range of 300–800 nm at a scan rate of 600 nm/min, with air used as the reference. Percentage transmittance values were averaged from three independent samples.

### 2.6. Mechanical Properties

Mechanical properties of the films, including tensile strength (TS), elongation at break (EB), and Young’s modulus (YM), were determined using a universal testing machine (Instron 5965, Instron, Norwood, MA, USA) in accordance with ASTM D882-09. The films were cut into rectangular strips with dimensions of 10 cm × 2.5 cm and tested along both the machine direction (MD) and cross direction (CD). Film thickness was measured using a digital micrometer (Mitutoyo, Kawasaki, Japan) at five different positions on each specimen, and the average value was reported. Tensile measurements were conducted at a crosshead speed of 500 mm/min with an initial grip separation of 5 cm. Prior to testing, all samples were conditioned at 25 °C and 50% relative humidity for 48 h. Mechanical properties were calculated as the average of fifteen replicate specimens for each formulation.

### 2.7. Water Vapor Permeability

Water vapor permeability (WVP) of the films was determined using the standard cup method in accordance with ASTM E96/E96M-12, “*Standard Test Methods for Water Vapor Transmission of Materials*.” [25]. Film samples were cut into circular specimens with a diameter of 7 cm and sealed onto aluminum permeability cups containing silica gel as the desiccant. An O-ring and molten paraffin wax were used to ensure an airtight seal between the film and the cup. The assembled cups were placed in a controlled humidity chamber (Binder KBF 720, Binder GmbH, Tuttlingen, Germany) maintained at 25 ± 2 °C and 50 ± 2% relative humidity. The cups were weighed periodically until a constant weight change rate was achieved. WVP measurements were conducted in quintuplicate for each formulation. Water vapor permeability was calculated using Equation (1):

(1) WVP = (WVTR × L)/ΔP

where WVTR is the water vapor transmission rate (g·m^−2^·h^−1^), obtained from the slope of the linear portion of the weight gain versus time curve (R^2^ > 0.99); L is the average film thickness (mm); and ΔP is the water vapor pressure difference (kPa) across the film.

### 2.8. Antimicrobial Activity

The antimicrobial activity of the films against *Staphylococcus aureus* ATCC 25923 was evaluated using a broth dilution assay. The bacterial inoculum was prepared in nutrient broth (HiMedia M096, HiMedia Laboratories Private Limited, Maharashtra, India) to achieve an initial concentration of approximately 5.3 log CFU/mL. Prior to testing, film samples were UV-sterilized and cut into strips (1 g). Each film sample was immersed in 9 mL of sterile 0.1% peptone solution containing 1 mL of the bacterial inoculum and incubated at 37 °C for 24 h under static conditions. Following incubation, serial dilutions (10^−1^ to 10^−6^) were prepared and plated onto nutrient agar. The plates were incubated at 37 °C for an additional 24 h, after which viable colonies were enumerated. Antimicrobial efficacy was expressed as the logarithmic reduction in viable cell counts relative to the control sample without film contact, and results were reported as log CFU/mL.

### 2.9. Statistical Analysis

Experimental data were analyzed using one-way analysis of variance (ANOVA) performed with IBM SPSS Statistics software (version 22; IBM Corp., Armonk, NY, USA). Differences among mean values were evaluated using Duncan’s multiple range test, with statistical significance set at *p* < 0.05.

## 3. Results and Discussion

### 3.1. Proton Nuclear Magnetic Resonance (^1^H-NMR) Analysis

**Figure 2:**
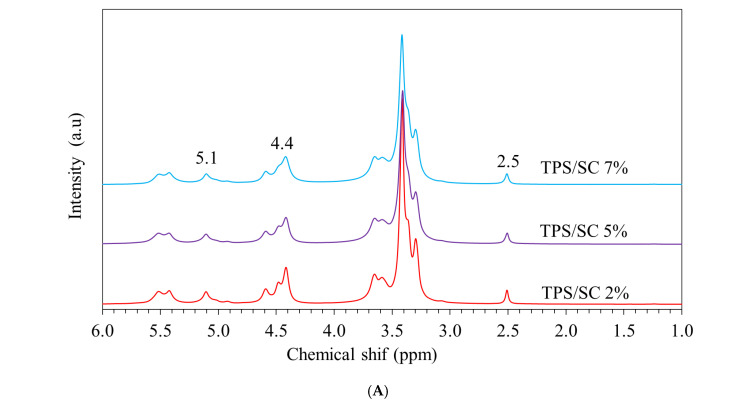

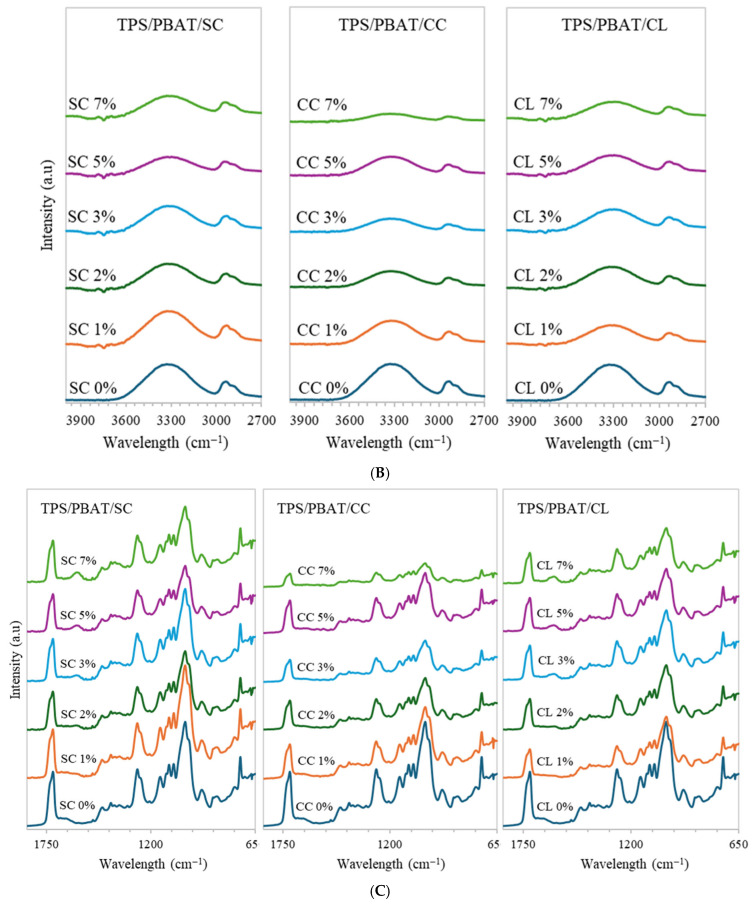
(**A**) ^1^H NMR spectra of TPS incorporated with sodium citrate (SC) as a representative organic salt at 2, 5, and 7 wt%. and FTIR spectra of TPS/PBAT films containing sodium citrate (SC), calcium citrate (CC), and calcium lactate (CL) at 1, 2, 3, 5, and 7 wt% in the range of (**B**) 2700–4000 cm^−1^ and (**C**) 650–1750 cm^−1^.

The ^1^H NMR spectra of thermoplastic starch (TPS) containing sodium citrate (SL) at 2, 5, and 7 wt% (Figure 2A) displayed characteristic resonances associated with the anhydroglucose unit (AGU) structure of starch. Signals in the range of approximately 3.2–4.0 ppm were attributed to the H2–H6 protons along the starch backbone, while the resonance observed at around 5.1–5.3 ppm corresponded to the anomeric proton (H1) of the α-glucopyranose unit, in agreement with previous reports [2,6,26]. These features indicate that the fundamental polysaccharide structure of starch remained intact after extrusion. No additional resonances indicative of covalent interactions, such as ester bond formation between starch hydroxyl groups and citrate carboxyl groups, were detected. Furthermore, the position of the anomeric proton signal and the resonances related to hydroxyl-bearing carbons showed no significant chemical shift changes when compared with neat TPS. This spectral consistency suggests that sodium citrate does not chemically react with starch chains under the processing conditions applied. Instead, the absence of peak shifts or new signals supports the role of sodium citrate as a modifier of hydrogen bonding within the TPS matrix rather than as a reactive crosslinking agent. Its presence is likely associated with non-covalent interactions that weaken intermolecular hydrogen bonding between starch chains, thereby promoting chain separation and increased molecular mobility. It should be noted that TPS samples incorporated with calcium-based salts (calcium citrate and calcium lactate) were not subjected to ^1^H NMR analysis. This is primarily due to their limited solubility in DMSO-d_6_ and the presence of divalent Ca^2+^ ions, which can induce strong ionic interactions and aggregation within the starch matrix. These effects hinder complete dissolution and result in broadened or poorly resolved NMR signals, making reliable spectral interpretation difficult. Therefore, the molecular interactions in calcium-containing systems were instead evaluated using complementary techniques such as FTIR and SEM analyses. These approaches confirmed the role of calcium salts in promoting ionic crosslinking and modifying the TPS/PBAT matrix. Overall, the ^1^H NMR results indicate that sodium citrate is physically incorporated into the TPS network, contributing to enhanced flexibility and processability of the TPS/PBAT composite system at the molecular level.

### 3.2. Fourier-Transform Infrared Spectroscopy (FTIR)

The FTIR spectra of all samples, including TPS/PBAT films with and without organic salt incorporation, are presented in Figure 2B,C. Changes in band intensity and peak position mainly reflect variations in intermolecular interactions rather than the formation of new covalent bonds. In the hydroxyl stretching region (3200–3600 cm^−1^; Figure 2B), the broad absorption band corresponds to O–H stretching vibrations of hydroxyl groups in starch, as well as absorbed moisture [1,15,27]. Upon incorporation of active compounds, noticeable changes in band intensity and width were observed. Films containing citrate, particularly those associated with calcium ions, exhibited a slightly narrower O–H band with reduced intensity. This behavior suggests restricted mobility of some hydroxyl groups due to enhanced intermolecular interactions, likely arising from multidentate coordination between citrate carboxylate groups and hydroxyl groups along starch chains [1,27,28]. Such interactions are consistent with the formation of ionic associations and strengthened hydrogen bonding within the TPS phase [1,14].

In contrast, films containing lactate showed a broader O–H band with increased intensity, indicating greater heterogeneity of hydrogen bonding and a higher availability of free hydroxyl groups. This observation supports the plasticizing role of lactate, which disrupts native starch–starch hydrogen bonding and increases chain mobility [19,29]. A similar trend was observed in the carbonyl stretching region at 1710–1730 cm^−1^, attributed to the C=O stretching vibration of ester groups in PBAT. This band was present in all TPS/PBAT composite films. The incorporation of calcium citrate led to a gradual decrease in peak intensity with increasing citrate content, suggesting changes in interfacial compatibility between TPS and PBAT. Such interactions may influence the local environment of PBAT ester groups without inducing esterification reactions within the polymer backbone [1,3].

The fingerprint region (Figure 2C) further supports these interpretations. Additional absorption bands in the range of 1550–1650 cm^−1^ were detected in films containing citrate and lactate salts, corresponding to the asymmetric stretching vibration of carboxylate groups (–COO^−^). Citrate-containing films exhibited stronger and more distinct bands in this region than lactate-containing films, reflecting a higher density of carboxylate groups and stronger coordination interactions. The pronounced carboxylate signals in citrate systems, particularly in the presence of divalent calcium ions, indicate the formation of ionic crosslinking points within the starch matrix [14,30]. In contrast, lactate-containing films showed weaker carboxylate absorption, consistent with predominantly monodentate interactions and limited coordination capability. In the region between 1000 and 1150 cm^−1^, associated with C–O and C–O–C stretching vibrations of glycosidic bonds in starch, moderate changes in peak intensity were observed among the samples. Citrate-containing films displayed slightly sharper peaks, suggesting a more organized molecular environment within the starch phase [14,31,32]. Conversely, lactate-containing films exhibited broader peaks in this region, indicative of increased structural disorder in starch chains, which may influence mechanical properties and strength, as discussed later. Overall, the FTIR results confirm that the incorporation of citrate and lactate salts into TPS/PBAT films does not result in new covalent bond formation. Instead, the observed spectral changes reflect differences in the nature and strength of intermolecular interactions within the starch matrix. Citrate, particularly in combination with calcium ions, promotes stronger ionic interactions and hydrogen bonding, whereas lactate primarily acts as a plasticizer by enhancing molecular mobility and hydrogen bond heterogeneity.

### 3.3. Scanning Electron Microscopy (SEM)

**Figure 3:**
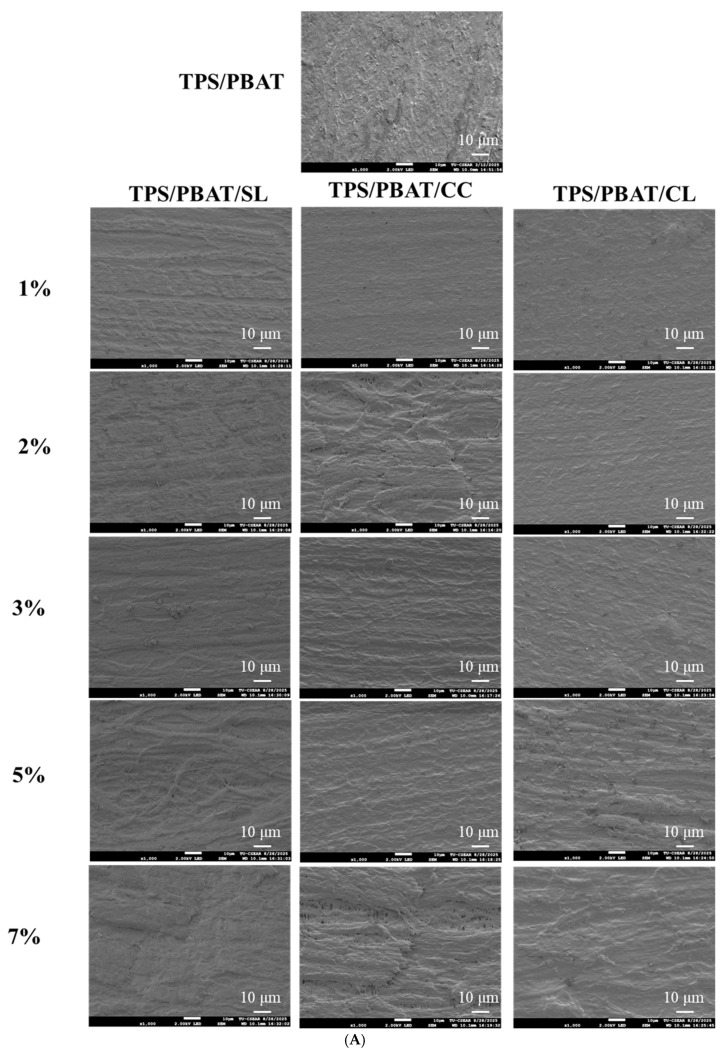

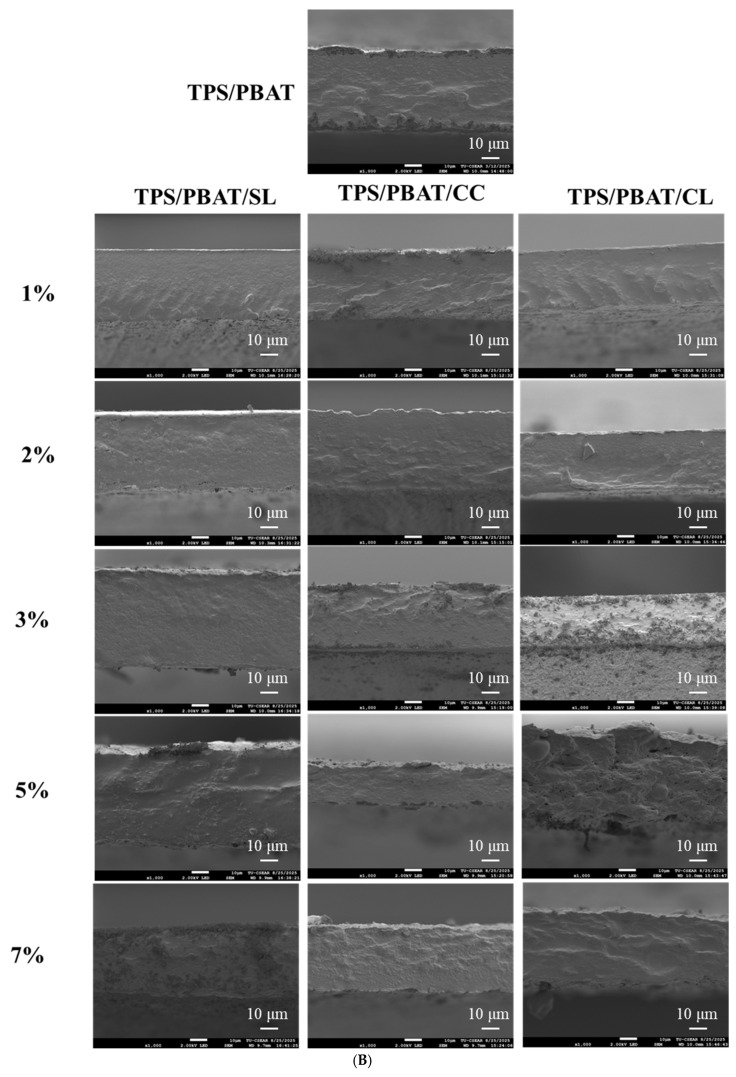
Microstructure of TPS/PBAT films containing sodium citrate (SC), calcium citrate (CC), and calcium lactate (CL) at 1, 2, 3, 5, and 7 wt%, including (**A**) surface morphology and (**B**) cross-section at magnification 1000×.

SEM micrographs revealed pronounced differences in the surface and cross-sectional morphologies of TPS/PBAT films containing different organic salts, as shown in Figure 3A,B, respectively. In general, all films exhibited relatively rough surface morphologies. This phenomenon can be primarily attributed to the intrinsic incompatibility between the hydrophilic TPS phase and the hydrophobic PBAT matrix. During melt processing, complete miscibility between these two components is difficult to achieve, leading to microphase separation and interfacial irregularities that manifest as surface roughness. In addition, the presence of glycerol as a plasticizer increases polymer chain mobility, which may promote surface undulations during film formation. Residual starch granules and non-uniform dispersion of components may also contribute to the heterogeneous surface structure. Despite this general roughness, distinct morphological variations were observed depending on the type of organic salt incorporated, which are consistent with the molecular-level changes identified by NMR and FTIR analyses. Films incorporated with calcium citrate exhibited smoother and more homogeneous surfaces without visible cracks or large voids. The corresponding cross-sections appeared dense and continuous, indicating efficient polymer chain packing and strong interfacial integrity [7,13,33].

This compact morphology is consistent with the ^1^H NMR results, which suggested restricted starch chain mobility due to interactions between citrate carboxylate groups and starch hydroxyl groups. Similarly, FTIR analysis of citrate-containing films showed reduced intensity and narrowing of the O–H stretching band, indicating a decrease in free hydroxyl groups and the formation of stronger hydrogen bonding and ionic interactions. These spectroscopic features support the formation of a dense and uniform microstructure [6,13]. In contrast, films containing lactate exhibited rougher and more heterogeneous surfaces, accompanied by the presence of microvoids and phase separation in the cross-sectional images. This morphology reflects a looser internal structure and reduced interfacial cohesion between the TPS and PBAT phases. NMR analysis of lactate-containing systems indicated increased starch chain mobility, resulting from the disruption of native hydrogen bonding. Consistently, FTIR spectra showed broader and more intense O–H stretching bands, suggesting a higher population of free hydroxyl groups and greater heterogeneity in hydrogen bonding interactions. A clear distinction was observed between sodium lactate and calcium lactate systems. TPS/PBAT films containing sodium lactate displayed pronounced surface roughness, evident microvoids, and clear phase separation in the cross-section, indicating limited interfacial adhesion between starch and PBAT [13,14]. In contrast, films containing calcium lactate exhibited smoother and more continuous surfaces, with denser cross-sectional structures and substantially reduced interfacial gaps. This improvement in morphology can be attributed to the role of Ca^2+^ ions in forming ionic bridges within the starch matrix, which enhances structural organization and interfacial stability [19,34]. These observations are consistent with FTIR results showing narrowing of the O–H stretching band and NMR data indicating reduced polymer chain mobility. Overall, the SEM observations support the conclusions drawn from spectroscopic analyses. Sodium lactate primarily acts as a plasticizer, increasing molecular mobility and promoting microstructural heterogeneity. In contrast, calcium lactate facilitates the formation of an ionic network within the starch phase, leading to improved microstructural integrity and enhanced phase compatibility in the TPS/PBAT system.

### 3.4. Light Transmission

**Figure 4:**
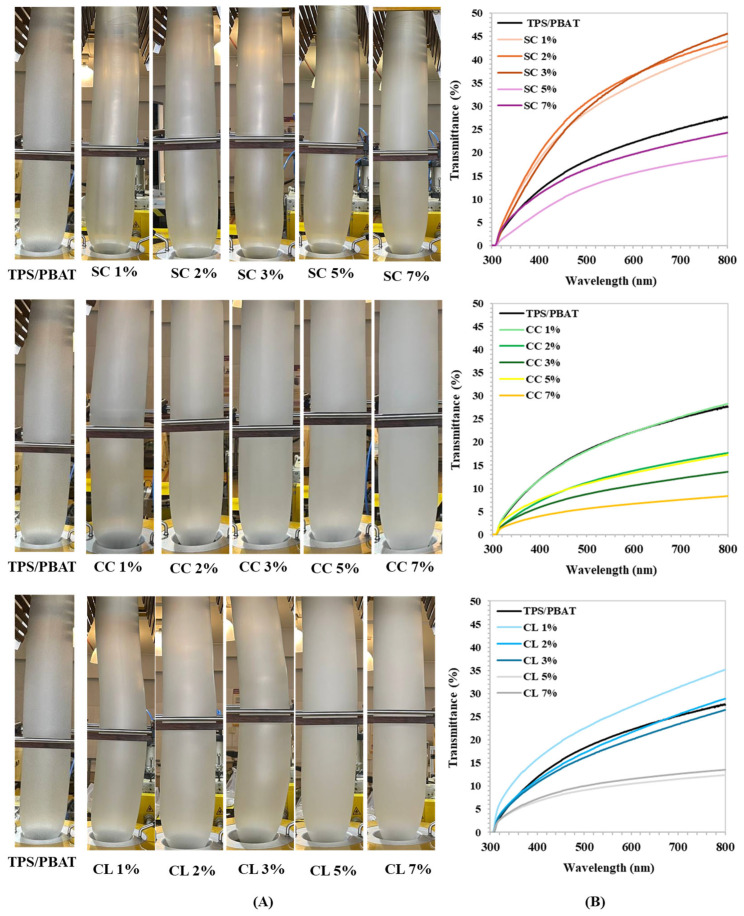
(**A**) Appearance during the blown-film extrusion process and (**B**) transparency of TPS/PBAT films containing sodium citrate (SC), calcium citrate (CC), and calcium lactate (CL) at 1, 2, 3, 5, and 7 wt%.

The visual appearance of TPS/PBAT films and films incorporated with SC, CC, and CL at different concentrations revealed clear differences in transparency and surface uniformity, as shown in Figure 3. The appearance of TPS/PBAT during blown-film extrusion is presented in Figure 4A. The control TPS/PBAT film exhibited a semi-transparent appearance with a relatively smooth and homogeneous surface [6]. At low additive contents (1–3 wt%), the films largely retained good structural continuity and surface uniformity. In contrast, increasing the additive concentration to 5–7 wt% resulted in a noticeable increase in film opacity, indicating enhanced light scattering caused by internal structural heterogeneity or microphase aggregation.

UV–visible transmittance spectra (Figure 4B) were consistent with these visual observations. The control TPS/PBAT film showed a gradual increase in light transmittance with increasing wavelength across the range of 300–800 nm. Films containing SC exhibited higher transmittance in the visible region than the control, particularly at concentrations of 1–3 wt%, suggesting good additive dispersion and a relatively uniform film structure. However, further increasing the SC content to 5–7 wt% led to a pronounced reduction in transmittance, which is consistent with the observed increase in film turbidity. Films containing CC and CL generally exhibited lower light transmittance than SC-containing films at equivalent concentrations. This reduction was especially evident in the ultraviolet region (300–400 nm), indicating enhanced UV-shielding capability. The reduced transmittance can be attributed to a combination of intrinsic UV absorption by the molecular structures of the additives and increased light scattering resulting from structural heterogeneity at higher loadings [13,15]. Overall, these results demonstrate that both the type and concentration of organic salt additives play a critical role in balancing optical transparency and light-protection performance, which is an important consideration for packaging applications involving light-sensitive food products.

### 3.5. Mechanical Properties

**Figure 5:**
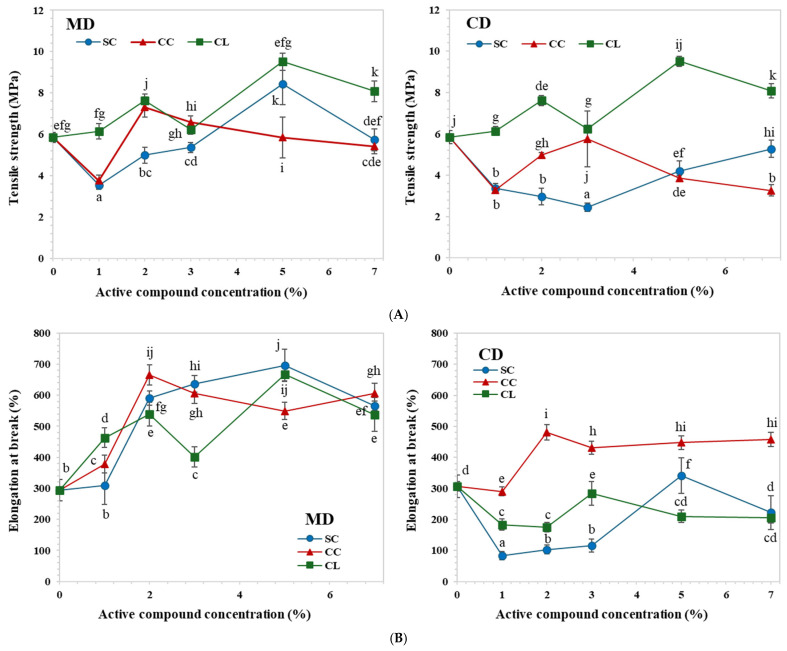

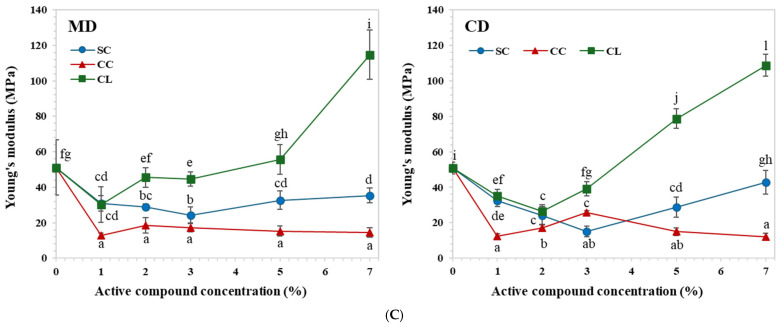
Mechanical properties of TPS/PBAT films containing sodium citrate (SC), calcium citrate (CC), and calcium lactate (CL) at 1, 2, 3, 5, and 7 wt%, including (**A**) tensile strength, (**B**) elongation at break, and (**C**) Young’s modulus. MD and CD refer to the machine and cross directions, respectively. *n* = 10; values with different statistical letters indicate significant differences at α = 0.05.

Figure 5A presents the tensile strength of TPS/PBAT films incorporated with different organic salts. The tensile strength showed a pronounced dependence on both the type and concentration of the additives. Incorporation of SC at low concentrations (1–3 wt%) resulted in tensile strength values that were comparable to or slightly higher than those of the control film. This behavior can be attributed to structural reinforcement arising from hydrogen-bonding interactions between the carboxylate groups of the salt and hydroxyl groups of starch, which enhance matrix uniformity and interfacial adhesion between the TPS and PBAT phases [5,17,35]. In contrast, further increasing the SC content to 5–7 wt% led to a reduction in tensile strength, likely due to additive aggregation or increased internal heterogeneity that acts as stress concentration sites under tensile loading [1,14]. Films containing CC and CL generally exhibited lower tensile strength than those containing sodium salts at equivalent concentrations. In particular, higher CC contents resulted in a more pronounced decrease in tensile strength. This behavior may be associated with the formation of localized ionic crosslinks between Ca^2+^ ions and starch hydroxyl groups, which increase structural rigidity but promote brittleness and reduce the efficiency of stress transfer within the polymer matrix [11,17].

The elongation at break of TPS/PBAT films is shown in Figure 5B. The control film exhibited relatively high elongation, reflecting the intrinsic flexibility of PBAT. Upon incorporation of organic salts, elongation at break initially increased, indicating a partial plasticizing effect of the additives that promotes structural continuity within the TPS phase [5,6,35]. However, increasing the SC content to 5–7 wt% resulted in a significant decrease in elongation, which is consistent with SEM observations showing increased structural heterogeneity and microcrack formation. For films containing calcium citrate and calcium lactate, elongation at break decreased markedly at concentrations up to 3 wt%, reflecting restricted polymer chain mobility due to strong ionic interactions involving Ca^2+^ ions. Interestingly, at higher loadings (5–7 wt%), elongation at break increased again, suggesting that excess salt may promote plasticization and improve phase compatibility between TPS and PBAT, thereby enhancing deformability [30,32].

Figure 5C shows the Young’s modulus of TPS/PBAT films. The modulus values indicate a balance between stiffness and flexibility in the control system. Upon addition of organic salts, Young’s modulus generally increased with increasing additive concentration, particularly in films containing calcium lactate [13,33]. These films exhibited significantly higher modulus values than those containing sodium citrate or calcium citrate, reflecting enhanced stiffness associated with ionic crosslink formation within the TPS matrix. This trend is consistent with FTIR results showing changes in the O–H stretching region, which indicate modification of the hydrogen-bonding network and stronger intermolecular interactions in calcium-containing systems.

### 3.6. Water Vapor Permeability

**Figure 6:**
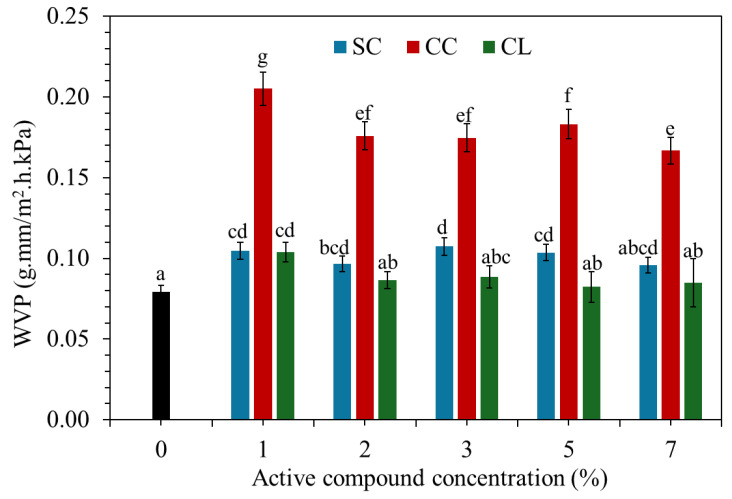
Water vapor permeability (WVP) of TPS/PBAT films containing sodium citrate (SC), calcium citrate (CC), and calcium lactate (CL) at 1, 2, 3, 5, and 7 wt%. The black bar represents the control film (TPS/PBAT without organic salt). Values are expressed as mean ± standard deviation (n = 5). Different letters indicate significant differences at α = 0.05.

Figure 6 presents the water vapor permeability (WVP) of TPS/PBAT films incorporated with organic salts, including SC, CC, and CL, at various concentrations. For SC-containing films, WVP showed a slight increase compared with the control, particularly at low concentrations (1–3 wt%). This behavior can be attributed to the hydrophilic nature of sodium citrate, which disrupts the hydrogen-bonding network of starch and promotes a more open matrix structure, thereby facilitating water vapor diffusion [19,30,34]. However, when the SC content was increased to 5–7 wt%, the WVP values tended to stabilize or slightly decrease. This trend may be associated with improved structural organization and enhanced compatibility between the TPS and PBAT phases at higher additive loadings. In contrast, films containing calcium citrate exhibited significantly higher WVP values than those containing SC and CL at all concentrations, with the highest WVP observed at 1 wt% CC. This behavior may result from non-uniform ionic crosslinking between Ca^2+^ ions and starch hydroxyl groups, leading to a rigid but discontinuous microstructure with the formation of microvoids [8,9]. Such features can provide preferential pathways for water vapor transport, which is consistent with SEM observations showing internal structural discontinuities in CC-containing systems. Among all formulations, films incorporated with calcium lactate showed the lowest WVP values and only minor changes with increasing additive concentration. These results suggest that calcium lactate effectively promotes the formation of a denser and more homogeneous matrix structure [7,13,33]. The combined effects of ionic interactions involving Ca^2+^ ions and the relatively small molecular size of the lactate moiety appear to restrict water vapor diffusion through the film [13,16]. Overall, the WVP results demonstrate that both the metal ion type and the molecular structure of the organic salts play critical roles in governing water vapor transport in TPS/PBAT films. Sodium-based salts tend to increase water vapor permeability, whereas calcium-based salts, particularly calcium lactate, are more effective in enhancing the water vapor barrier performance. This behavior is consistent with the observed microstructural features of the films.

### 3.7. Antimicrobial Activity

**Figure 7:**
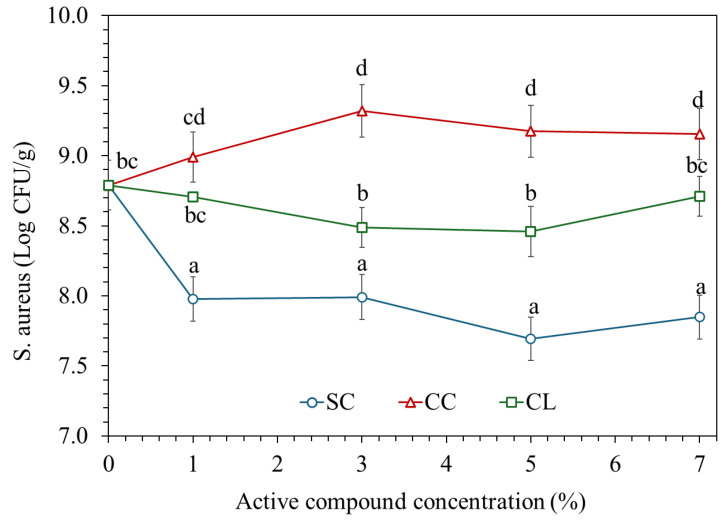
Antimicrobial activity of TPS/PBAT films containing sodium citrate (SC), calcium citrate (CC), and calcium lactate (CL) at 1, 2, 3, 5, and 7 wt% against *Staphylococcus aureus*. n = 4; values with different statistical letters indicate significant differences at α = 0.05.

Figure 7 illustrates the inhibitory effect of TPS/PBAT films incorporated with SC, CC, and CL at concentrations ranging from 0 to 7 wt%, evaluated based on the remaining *S. aureus* counts (log CFU/g) after testing. The neat TPS/PBAT matrix without organic salts exhibited negligible antimicrobial activity, indicating that the observed antibacterial effects are primarily attributed to the incorporated organic salts rather than the polymer matrix itself. The polymer matrix mainly acts as a carrier and controls the release of active compounds. TPS/PBAT films containing SC exhibited a pronounced reduction in *S. aureus* populations at all concentrations. This result reflects the strong antimicrobial effectiveness of SC, which may be associated with localized pH reduction at the film surface and disruption of ionic balance within bacterial cells, potentially affecting membrane integrity and cellular function [10,36]. In addition, organic acid salts such as citrate can act in their undissociated form, allowing them to penetrate bacterial cell membranes and dissociate intracellularly, leading to cytoplasmic acidification and disruption of essential metabolic processes. Films containing CL showed a moderate inhibitory effect. The *S. aureus* population decreased with increasing additive concentration up to 3–5 wt%, followed by a slight increase at 7 wt%. This behavior may reflect a balance between the release of active species and increased matrix density at higher salt loadings, which can restrict the diffusion of antimicrobial agents to the film–microorganism interface [6,30]. In contrast, films incorporated with CC exhibited the lowest antimicrobial activity. The *S. aureus* counts tended to increase with increasing CC concentration, particularly in the range of 3–7 wt%. This trend may be attributed to strong ionic interactions between Ca^2+^ ions and the starch matrix, which reduce the availability and release of free carboxylate groups, thereby limiting antimicrobial efficacy [5,18]. Furthermore, the antimicrobial performance is closely related to the microstructure of the films, which governs the release behavior of active compounds. Films with more heterogeneous and porous structures may facilitate faster diffusion of active species, whereas more compact matrices can restrict their release. These observations are consistent with the SEM results, where lactate-containing films exhibited more heterogeneous morphologies, while citrate-based systems showed relatively denser and more uniform structures. Therefore, TPS/PBAT films containing SC demonstrated the highest inhibitory activity against *S aureus*, followed by CL, while CC showed the lowest antimicrobial performance. These findings indicate that both the metal ion type and the release behavior of active species, governed by polymer–additive interactions and film microstructure, play critical roles in determining the antimicrobial properties of bio-based packaging films.

## 4. Conclusions

The incorporation of different organic salts, namely SC, CC, and CL, at varying concentrations (1, 2, 3, 5, and 7 wt%) into TPS/PBAT films plays a critical role in tailoring their molecular structure and functional properties through distinct interaction mechanisms. SC primarily promotes hydrogen-bonding interactions with starch, leading to a more homogeneous matrix structure, improved stress transfer, and the highest antimicrobial activity against *Staphylococcus aureus*. In contrast, CL and CC tend to induce ionic crosslinking through Ca^2+^ ions, which increases structural density and Young’s modulus but restricts polymer chain mobility and limits the release of active species, particularly in CC-containing systems. Structural analyses using NMR, FTIR, and SEM support clear structure–property relationships governing the mechanical performance, water vapor barrier properties, and optical behavior of the films, which can be effectively balanced by the appropriate selection of organic salt type and concentration. Overall, TPS/PBAT films incorporated with SC exhibit the greatest potential for the development of biodegradable active packaging with antimicrobial functionality, whereas CL-based systems are more suitable for applications requiring enhanced barrier performance and structural stiffness.

## Data Availability

The original contributions presented in the study are included in the article, further inquiries can be directed to the corresponding authors.
